# Dietary oxidative balance and renal impairment in diabetes identified by machine learning and functional analysis

**DOI:** 10.3389/fnut.2026.1792300

**Published:** 2026-05-04

**Authors:** Renjie Wang, Xincan Chen, Luheng Shen, Lei Wu, Zicheng Zhu, Zhihong Liu, Zhiwen Xie, Juntao Jiang

**Affiliations:** Department of Urology, Shanghai General Hospital, Shanghai Jiao Tong University School of Medicine, Shanghai, China

**Keywords:** albuminuria, diabetes, machine learning, oxidative balance, oxidative balance-related dietary factors, renal impairment

## Abstract

**Background:**

Oxidative stress has been implicated in albuminuria-related renal abnormalities in diabetes; however, the combined associations of pro-oxidant and antioxidant dietary components with UACR-defined renal impairment remain unclear. This study aimed to investigate the association between dietary oxidative balance and renal impairment in patients with diabetes and to conduct exploratory *in vitro* experiments examining whether selected nutrients modulate AKT phosphorylation under high-glucose conditions.

**Methods:**

Data from diabetic participants in the National Health and Nutrition Examination Survey (NHANES) 1999–2020 were analyzed. Based on the conceptual framework of the oxidative balance score (OBS), a panel of oxidative balance–related dietary components, including antioxidant and pro-oxidant factors, was selected for analysis. Multiple machine learning models were applied to assess associations between these dietary factors and renal impairment assessed by UACR, with model interpretability analyses identifying key contributors. *In vitro* experiments were conducted to explore whether selected nutrients modulated AKT phosphorylation under high-glucose conditions.

**Results:**

A total of 9,764 participants with diabetes were included. Among all models, the random forest algorithm achieved the best predictive performance. Feature importance analysis identified vitamin E, β-carotene, and magnesium as the most influential dietary factors associated with renal impairment in diabetes. In HK-2 cells, these nutrients partially restored AKT phosphorylation that was suppressed by high-glucose conditions, suggesting possible pathway-level relevance under high-glucose conditions.

**Conclusion:**

This study identified significant associations between oxidative balance-related dietary components and renal impairment in diabetes and highlighted vitamin E, β-carotene, and magnesium as key antioxidant-related dietary factors. Exploratory *in vitro* findings suggest that these nutrients may modulate high-glucose–induced AKT signaling suppression, warranting further investigation in future observational and mechanistic studies.

## 1 Introduction

Renal impairment in diabetes remains a common clinical concern. Despite advances in glycaemic and blood pressure control, renal abnormalities in diabetes remain prevalent, suggesting that factors beyond traditional risk factors may also contribute (1). Among the factors associated with renal abnormalities in diabetes, oxidative stress has received substantial attention (2, 3).

Oxidative stress refers to a pathological imbalance between pro-oxidant and antioxidant forces, favoring oxidative damage and contributing to inflammation and cellular injury (4). In the context of diabetes, chronic hyperglycaemia promotes excessive reactive oxygen species (ROS) production, impairs antioxidant defenses, and induces oxidative stress-related cellular disturbances (5). Excessive ROS generation under high-glucose conditions has been widely implicated in cellular stress and diabetes-related renal impairment (6). However, despite extensive mechanistic insights, current research has largely focused on individual oxidative markers or isolated pathways, leaving the overall oxidative environment insufficiently characterized.

The oxidative balance score (OBS) integrates multiple dietary and lifestyle factors, encompassing both pro-oxidant and antioxidant exposures, to reflect the overall oxidative environment (7). However, the specific components included in OBS and the corresponding scoring methods vary across studies, and no universally accepted standard has been established. Although individual antioxidant or pro-oxidant components have been linked to renal outcomes, their combined effects on renal impairment in patients with diabetes remain poorly defined (8). In the present study, we used the OBS framework as a conceptual basis to select oxidative balance-related dietary components from NHANES and examined their associations with UACR-defined renal impairment in patients with diabetes.

To address these gaps, this study utilized data from the National Health and Nutrition Examination Survey (NHANES) and applied machine learning algorithms to investigate the association between OBS-related dietary components and UACR-defined renal impairment in patients with diabetes. Compared with traditional statistical approaches, machine learning methods are better suited for capturing complex, non-linear relationships in high-dimensional health data (9, 10). Through multimodel comparison and SHapley Additive exPlanations (SHAP) analysis, this study aimed to identify key dietary factors associated with UACR-defined renal impairment and to establish an interpretable predictive framework. Furthermore, exploratory *in vitro* experiments were conducted to provide exploratory pathway-level context for the identified nutrients.

## 2 Methods

**Figure 1:**
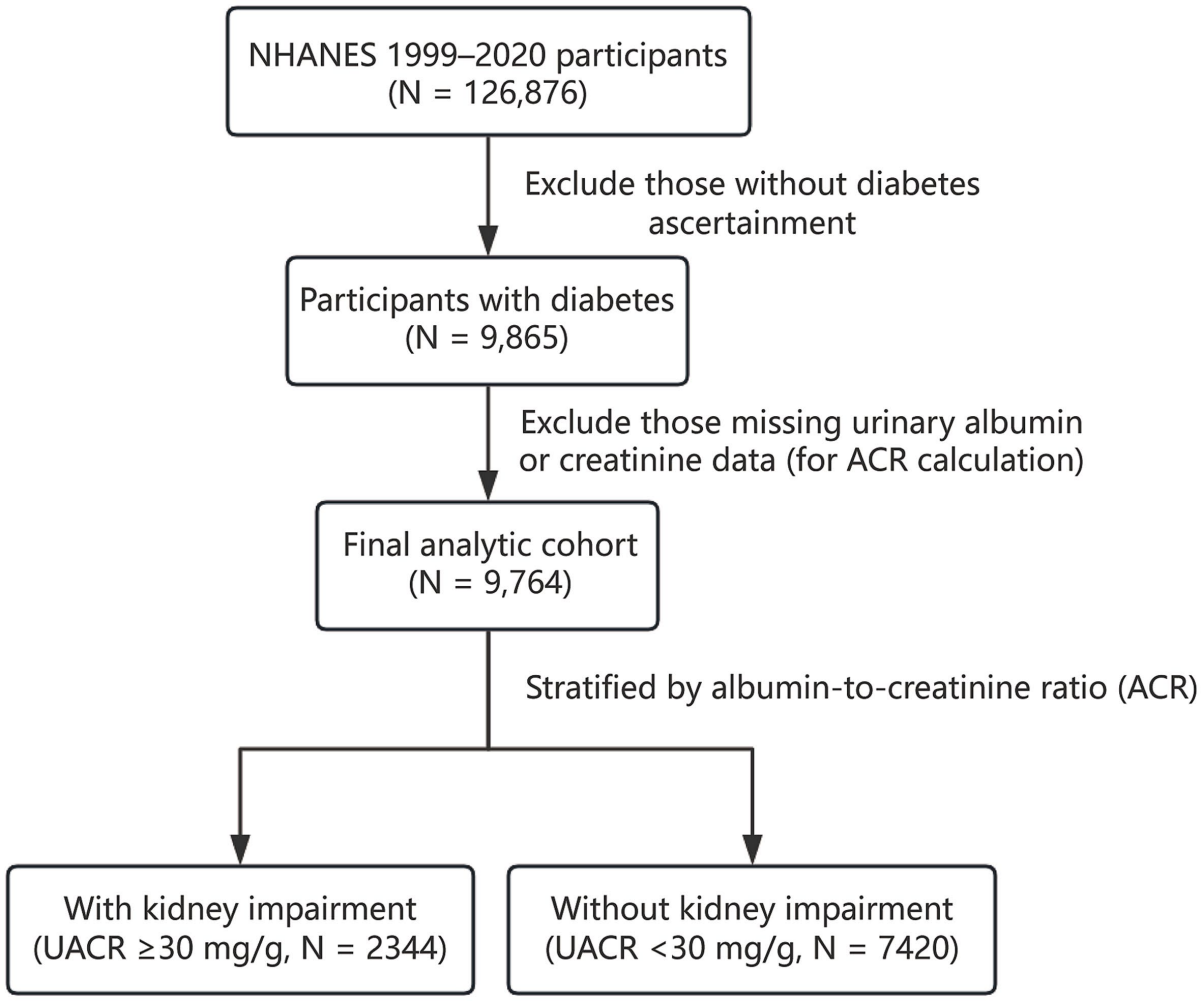
Participant flow diagram. Final analytic cohort of 9,764 diabetic participants from NHANES 1999–2020 for the main analysis, stratified by albumin-to-creatinine ratio (ACR) into groups with and without kidney impairment.

The data used in this study were derived from the National Health and Nutrition Examination Survey (NHANES), a continuous, open-access multipurpose survey established by the centers for Disease Control and Prevention (CDC). Participants from the 1999–2020 NHANES survey cycles were included if they had available information on selected oxidative balance-related dietary components, diabetes diagnosis, and UACR data, resulting in a final analytic cohort of 9,764 participants for the main analysis (Figure 1).

### 2.1 Selection of oxidative balance-related dietary components

Data on 19 oxidative balance-related variables were obtained from NHANES. Participants underwent 24-h dietary recall interviews in mobile examination centers, and data on oxidative balance-related variables, including antioxidant and pro-oxidant/exposure factors, were extracted for analysis.

### 2.2 Diabetes diagnosis and renal impairment assessment

A diabetes diagnosis was confirmed if participants reported being told by a physician or other health care professional that they had diabetes. Additionally, individuals meeting one or more of the following criteria were also classified as having diabetes: a glycated hemoglobin (HbA1c) concentration ≥6.5%, a fasting plasma glucose concentration ≥7 mmol/L, a 2-h plasma glucose concentration ≥11.1 mmol/L during an oral glucose tolerance test, a random plasma glucose concentration ≥11.1 mmol/L, or current use of antidiabetic medications.

Renal impairment was assessed using the urine albumin-to-creatinine ratio (UACR). Because albuminuria is widely recognized as an early marker of kidney damage, particularly in patients with diabetes, UACR was selected as the primary outcome indicator in this study. UACR was selected in this study to capture an albuminuria-based renal impairment phenotype in patients with diabetes. A UACR of 30–300 mg/g was considered moderately increased, whereas a UACR > 300 mg/g was considered severely increased. In this study, moderate or severe increases in the UACR were defined as indicators of renal impairment (11).

### 2.3 Baseline characteristics

Baseline characteristics, such as demographic, lifestyle, and health status variables, were collected. These included age, sex (male or female), race/ethnicity (Mexican American, other Hispanic, non-Hispanic White, non-Hispanic Black, and other categories), family poverty-to-income ratio (categorized as 0–1, 1–3, or >3), smoking status (never, former, or current), and the presence of hyperlipidaemia or hypertension. Data on age, sex, race/ethnicity, and the family poverty-to-income ratio were retrieved from the NHANES demographic data module, while information on smoking and physical activity was obtained from questionnaire data. Individuals who reported smoking fewer than 100 cigarettes in their lifetime were classified as never smokers, whereas others were categorized as former or current smokers on the basis of their response to the question, “Do you now smoke cigarettes?” Hyperlipidaemia and hypertension diagnoses were determined using laboratory measurements and self-reported data from the questionnaire module. Hyperlipidaemia was defined as high-density lipoprotein cholesterol levels less than 1.0 mmol/L in men or 1.3 mmol/L in women or triglyceride levels ≥1.8 mmol/L. Participants who reported using lipid-lowering medications or had been diagnosed with hyperlipidaemia or hypercholesterolemia by a physician were also classified as having hyperlipidaemia regardless of their lipid levels. Hypertension was defined as a mean systolic blood pressure ≥130 mmHg and/or a mean diastolic blood pressure ≥80 mmHg based on repeated blood pressure measurements obtained during the NHANES examination. Additionally, if participants answered “yes” to questions regarding antihypertensive medication use or a prior hypertension diagnosis, they were defined as having hypertension.

### 2.4 Statistical analysis for baseline characteristics

The statistical analysis was performed using the tableone package to generate tables of baseline characteristics for the study population. Continuous variables are presented as medians with interquartile ranges (IQRs), whereas categorical variables are expressed as frequencies and percentages. Comparative analyses were conducted using Pearson’s chi-square tests for categorical variables and Wilcoxon rank-sum tests for continuous variables. Table 1 summarizes the baseline characteristics of the final analytic cohort of 9,764 participants. For the machine learning analysis, participants with available UACR measurements were included. Variables with more than 20% missing values were excluded. Missing data handling for the machine learning models is described in Section 3.5.

**Table 1:** Baseline characteristics of diabetic participants according to UACR-defined renal impairment status.

Characteristic	Overall *N* = 9,764^1^	Diabetes without kidney impairment, *N* = 7,420^1^	Diabetes with kidney impairment, *N* = 2,344^1^	*p*-value^2^
BMI	30.70 (26.81, 35.70)	30.66 (26.85, 35.50)	30.90 (26.80, 35.94)	0.3
Missing	49	32	17	
Age	61.00 (49.00, 71.00)	60.00 (48.00, 69.00)	64.00 (54.00, 74.00)	<0.001
Sex				<0.001
Male	5,128 (53%)	3,818 (51%)	1,310 (56%)	
Female	4,636 (47%)	3,602 (49%)	1,034 (44%)	
Eth				<0.001
Non-Hispanic White	4,133 (42%)	3,211 (43%)	922 (39%)	
Non-Hispanic Black	2,157 (22%)	1,578 (21%)	579 (25%)	
Mexican American	1,767 (18%)	1,307 (18%)	460 (20%)	
Other Hispanic	849 (8.7%)	663 (8.9%)	186 (7.9%)	
Other Race – Including Multi-Racial	858 (8.8%)	661 (8.9%)	197 (8.4%)	
PIR				<0.001
<1	2,041 (21%)	1,496 (20%)	545 (23%)	
1–3	4,395 (45%)	3,244 (44%)	1,151 (49%)	
>3	3,328 (34%)	2,680 (36%)	648 (28%)	
Edu				<0.001
Below high school	1,384 (14%)	985 (13%)	399 (17%)	
High school	3,821 (39%)	2,869 (39%)	952 (41%)	
Above high	4,546 (47%)	3,556 (48%)	990 (42%)	
Missing	13	10	3	
Smoke				<0.001
Never	4,873 (50%)	3,791 (51%)	1,082 (46%)	
Former	3,234 (33%)	2,409 (32%)	825 (35%)	
Now	1,650 (17%)	1,217 (16%)	433 (19%)	
Missing	7	3	4	
Alcohol				<0.001
Never	1,438 (16%)	1,083 (16%)	355 (16%)	
Former	2,017 (22%)	1,425 (20%)	592 (27%)	
Mild	3,233 (35%)	2,503 (36%)	730 (33%)	
Moderate	1,103 (12%)	896 (13%)	207 (9.4%)	
Heavy	1,376 (15%)	1,056 (15%)	320 (15%)	
Missing	597	457	140	
Hypertension				<0.001
No	3,283 (34%)	2,813 (38%)	470 (20%)	
Yes	6,480 (66%)	4,607 (62%)	1,873 (80%)	
Missing	1	0	1	
Hyperlipidemia				<0.001
No	1,450 (15%)	1,171 (16%)	279 (12%)	
Yes	8,314 (85%)	6,249 (84%)	2,065 (88%)	

^1^Median (Q1, Q3); *n* (%).

^2^Wilcoxon rank sum test; Pearson’s Chi-squared test.

### 2.5 Machine learning

In this study, a machine learning–based analytical framework was applied to investigate the associations between oxidative balance-related variables and renal impairment in diabetes. During data preprocessing, participants with diabetes and available UACR measurements were first selected for analysis. Variables with more than 20% missing values were excluded from the dataset, and missing values in the remaining variables were handled using mean imputation within the model pipeline. Relevant features were then standardized as appropriate for specific models prior to model training. To reduce the risk of data leakage, imputation and scaling procedures were performed separately within each training fold during cross-validation.

The dataset was randomly divided into a training set (60%), a validation set (20%), and a test set (20%) using stratified sampling to maintain class distribution. Input features for the machine learning models included oxidative balance-related variables and clinical covariates. Supplementary Table 1 presents the oxidative balance-related dietary variables and related exposure variables included in the analysis. For model construction, five commonly used machine learning algorithms were systematically compared, including random forest, XGBoost, gradient boosting machine, logistic regression, and support vector machine (12). Hyperparameter tuning was performed for all models using grid search with stratified five-fold cross-validation on the training set. Model-specific probability thresholds were further optimized on the validation set. The final performance of each model was then assessed on the independent test set using the area under the receiver operating characteristic curve (AUC), precision-recall AUC (PR AUC), accuracy, sensitivity, specificity, F1 score, and balanced accuracy. In addition, 95% confidence intervals for AUC were estimated using bootstrap resampling, and calibration performance was assessed for the best-performing model. Model interpretability was assessed using SHapley Additive exPlanations (SHAP). SHAP values were calculated using the Python shap package, and the TreeExplainer method was applied to quantify the contribution of individual nutritional features to model predictions and to identify key dietary factors associated with renal outcomes. The machine learning models were trained and evaluated on the analytic dataset without incorporating NHANES survey weights, strata, or primary sampling units, as the primary aim was to identify and rank dietary factors associated with UACR-defined renal impairment within the study sample rather than to generate nationally representative estimates.

### 2.6 Identification of differentially expressed genes and enrichment analysis

To provide biological context for the dietary factors identified in the main analysis, we used the gene expression dataset GSE96804, which includes diabetic nephropathy and control kidney samples from GEO, as an external reference for exploratory enrichment analysis. GSE96804 contains 61 human glomerular samples, including 41 diabetic nephropathy samples and 20 normal control samples, and was generated using the Affymetrix Human Transcriptome Array 2.0 platform (GPL17586). Differentially expressed genes (DEGs) were identified using standard normalization and statistical procedures, with |log₂Fold Change| > 1 and *p* < 0.05 applied as screening thresholds.

Potential target genes of vitamin E, magnesium, and β-carotene were retrieved from SwissTargetPrediction, ChEMBL, and the Comparative Toxicogenomics Database (CTD). These nutrient-related targets were intersected with the identified DEGs to obtain overlapping gene sets for subsequent functional analysis.

Gene Ontology (GO) functional enrichment and Kyoto Encyclopedia of Genes and Genomes (KEGG) pathway analyses were performed to explore the potential biological functions and pathways associated with these overlapping genes.

### 2.7 Cell culture

The human proximal tubular epithelial cell line HK-2 was purchased from the Shanghai Institute of Cell Biology, Chinese Academy of Sciences (Shanghai, China), and cultured in DMEM/F12 medium (HyClone, Logan, UT, United States) supplemented with 10% fetal bovine serum (FBS; Gibco, Grand Island, NY, United States) and 1% penicillin–streptomycin (Gibco, Grand Island, NY, United States) at 37 °C in a humidified incubator with 5% CO₂. Cells were exposed to 30 mM D-glucose (Sigma-Aldrich, St. Louis, MO, United States) for 48 h to establish the high-glucose model, while cells cultured in 5.5 mM glucose served as the normal-glucose control. Under high-glucose conditions, cells were treated with vitamin E (25 μM; MBC, China), β-carotene (10 μM; Psaitong, China), or magnesium sulfate (MgSO₄, 1 mM; Sigma-Aldrich, St. Louis, MO, United States) for 48 h to assess their effects on cellular signaling. Control cells received an equal volume of vehicle.

### 2.8 Western blot

Western blot analysis was performed to assess AKT phosphorylation in HK-2 cells under different treatment conditions. The cells were washed with PBS, and protein lysates were extracted using 1 × RIPA buffer (Beyotime, Suzhou, China) supplemented with protease inhibitors (Roche, Indianapolis, IN, United States). Protein concentrations were determined using a BCA protein assay kit (Thermo Scientific, MA, United States). Equal amounts of protein were separated by SDS-PAGE and transferred onto PVDF membranes (Millipore, Billerica, MA, United States). After blocking, the membranes were incubated with primary antibodies against phospho-AKT (Ser473) (Abways, Shanghai, China), pan-AKT (Sigma-Aldrich, St. Louis, MO, United States), and β-actin (Beyotime, Shanghai, China). Protein bands were visualized using an enhanced chemiluminescence detection system (NCM, Suzhou, China) and quantified using ImageJ software. Western blot experiments were independently repeated three times. The bands shown in the figures are representative of three independent experiments, and quantitative data used for statistical analysis were derived from three independent experiments (*n* = 3).

### 2.9 Statistical analysis for *in vitro* experiments

Experimental data are presented as mean ± SD. Comparisons among multiple groups were performed using one-way analysis of variance (ANOVA), followed by an appropriate *post hoc* multiple-comparison test. All experiments were independently repeated at least three times. A *p*-value < 0.05 was considered statistically significant.

### 2.10 Ethics approval

The NHANES study protocols were approved by the NCHS Research Ethics Review Board, and all participants provided written informed consent. As the NHANES data are publicly available and de-identified, no additional ethical approval was required for this study.

## 3 Results

### 3.1 Demographic characteristics

Baseline demographic and clinical characteristics of the study population are summarized in Table 1. Participants with renal impairment were older and had a higher prevalence of hypertension and hyperlipidaemia compared with those without renal impairment (all *p* < 0.001). Differences were also observed in sex distribution, race/ethnicity, socioeconomic status, smoking status, and alcohol consumption patterns between the two groups. In contrast, body mass index did not differ significantly between groups.

### 3.2 Feature correlation analysis and model performance comparison of dietary elements

**Figure 2:**
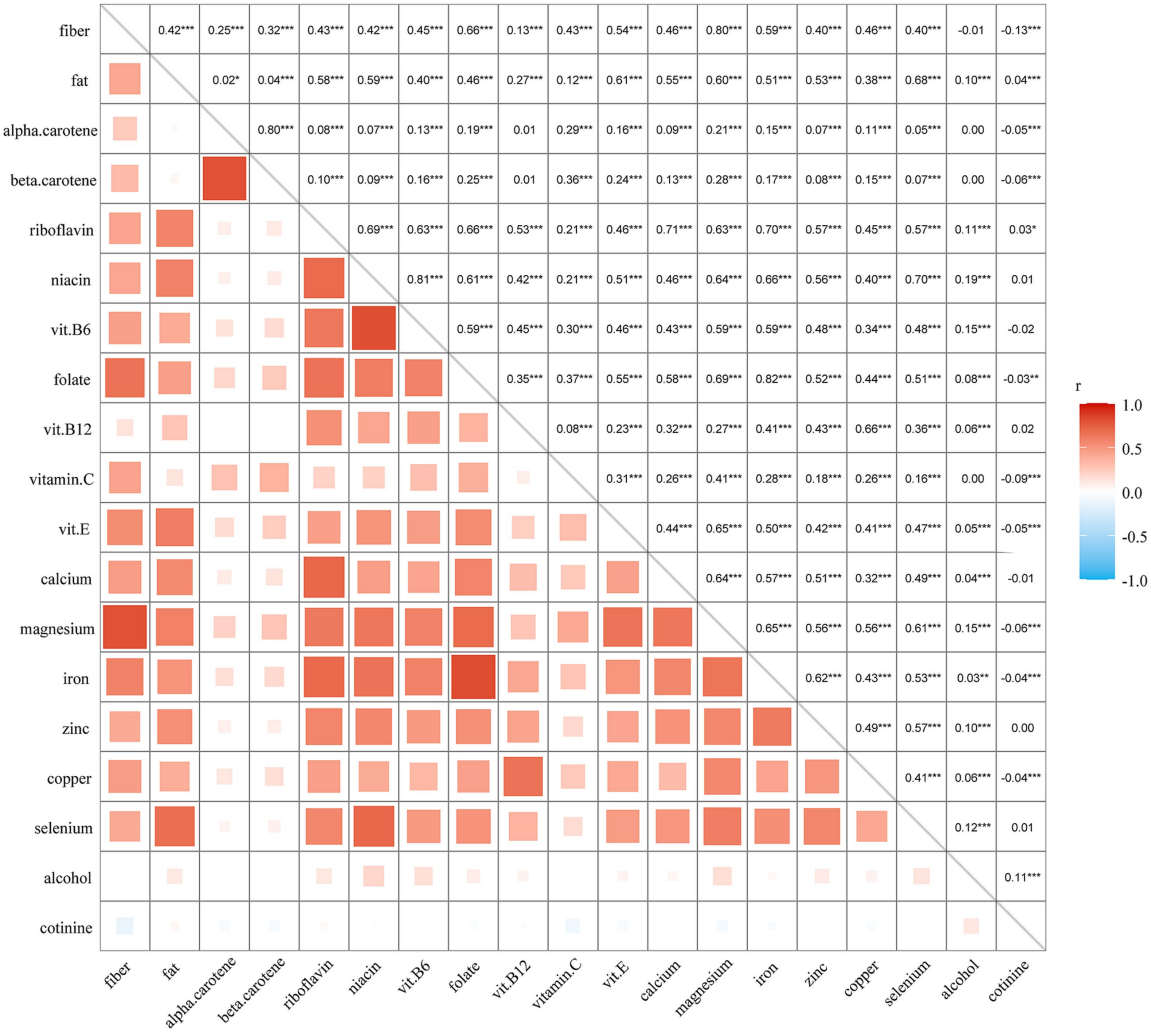
Correlation analysis of oxidative balance-related variables. Pairwise correlations among 19 oxidative balance-related variables included in the analysis are shown. *p < 0.05, **p < 0.01, ***p < 0.001.

Prior to model construction, correlations among oxidative balance-related variables were examined to assess feature relationships (Figure 2).

**Figure 3:**
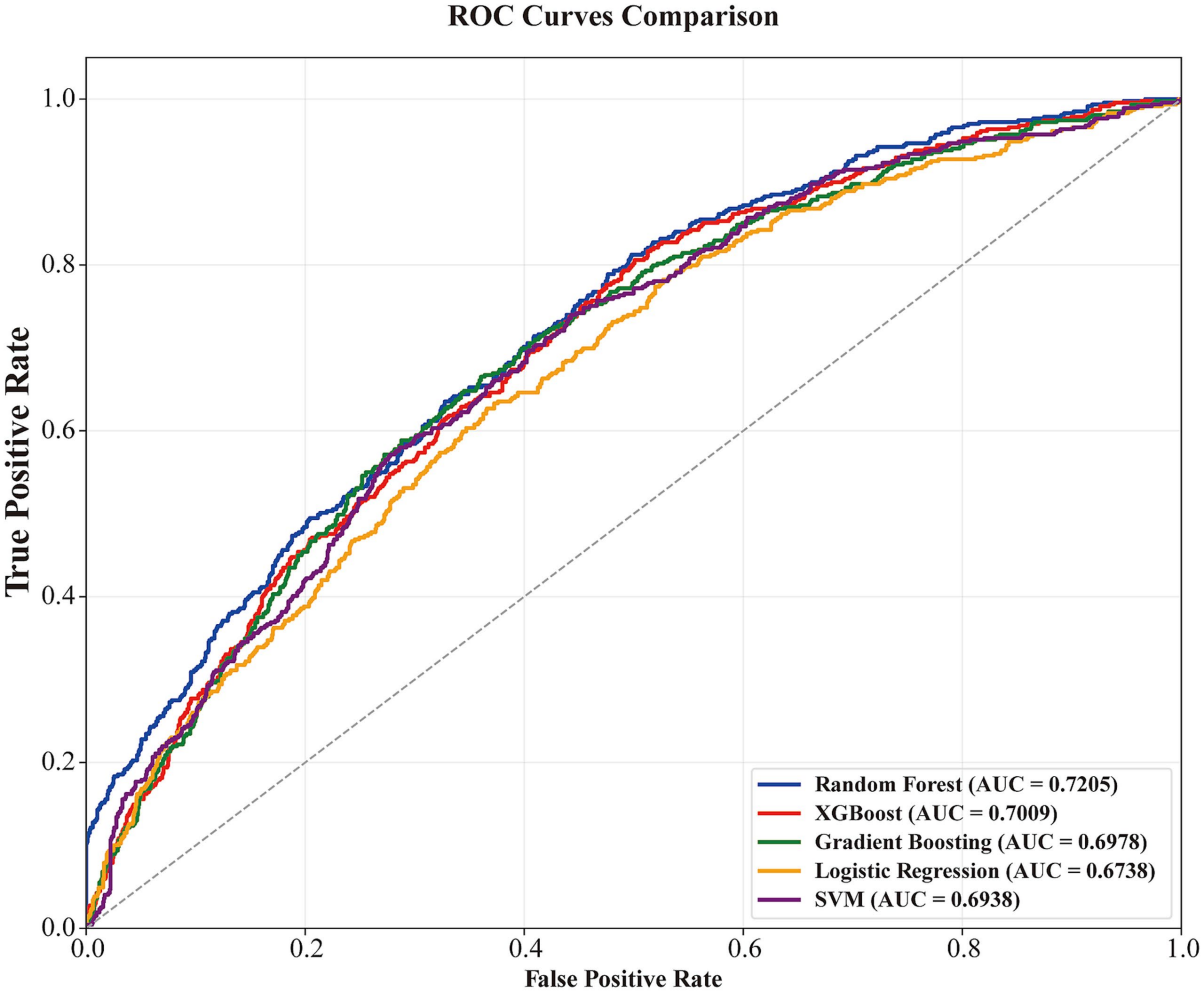
ROC curves of the five machine learning models in the test set. The ROC curves illustrate the predictive performance of random forest, XGBoost, gradient boosting, logistic regression, and support vector machine in classifying UACR-defined renal impairment.

The predictive performance of five machine learning models was subsequently compared (Figure 3 and Table 2). Among the evaluated algorithms, the random forest model demonstrated the best overall performance, achieving the highest AUC (0.7205, 95% CI: 0.6934–0.7461) and F1 score (0.4732). XGBoost and gradient boosting yielded similar intermediate performance. Support vector machine showed the highest specificity (0.7136), whereas logistic regression showed the highest sensitivity (0.7335) but the lowest AUC (0.6738). Detailed performance metrics are presented in Supplementary Table 3. The calibration performance of the random forest model is illustrated in Supplementary Figure 1.

**Table 2:** Predictive performance of five machine learning models in the test set.

Model	AUC (95% CI)	Sensitivity	Specificity	F1 Score
Random Forest	0.7205 (0.6934–0.7461)	0.7164	0.5856	0.4732
XGBoost	0.7009 (0.6739–0.7257)	0.6951	0.5923	0.4657
Gradient Boosting	0.6978 (0.6712–0.7240)	0.7100	0.5903	0.4723
SVM	0.6938 (0.6655–0.7194)	0.5778	0.7136	0.4652
Logistic Regression	0.6738 (0.6466–0.7008)	0.7335	0.5142	0.4485

### 3.3 SHAP value analysis of dietary influencing factors

**Figure 4:**
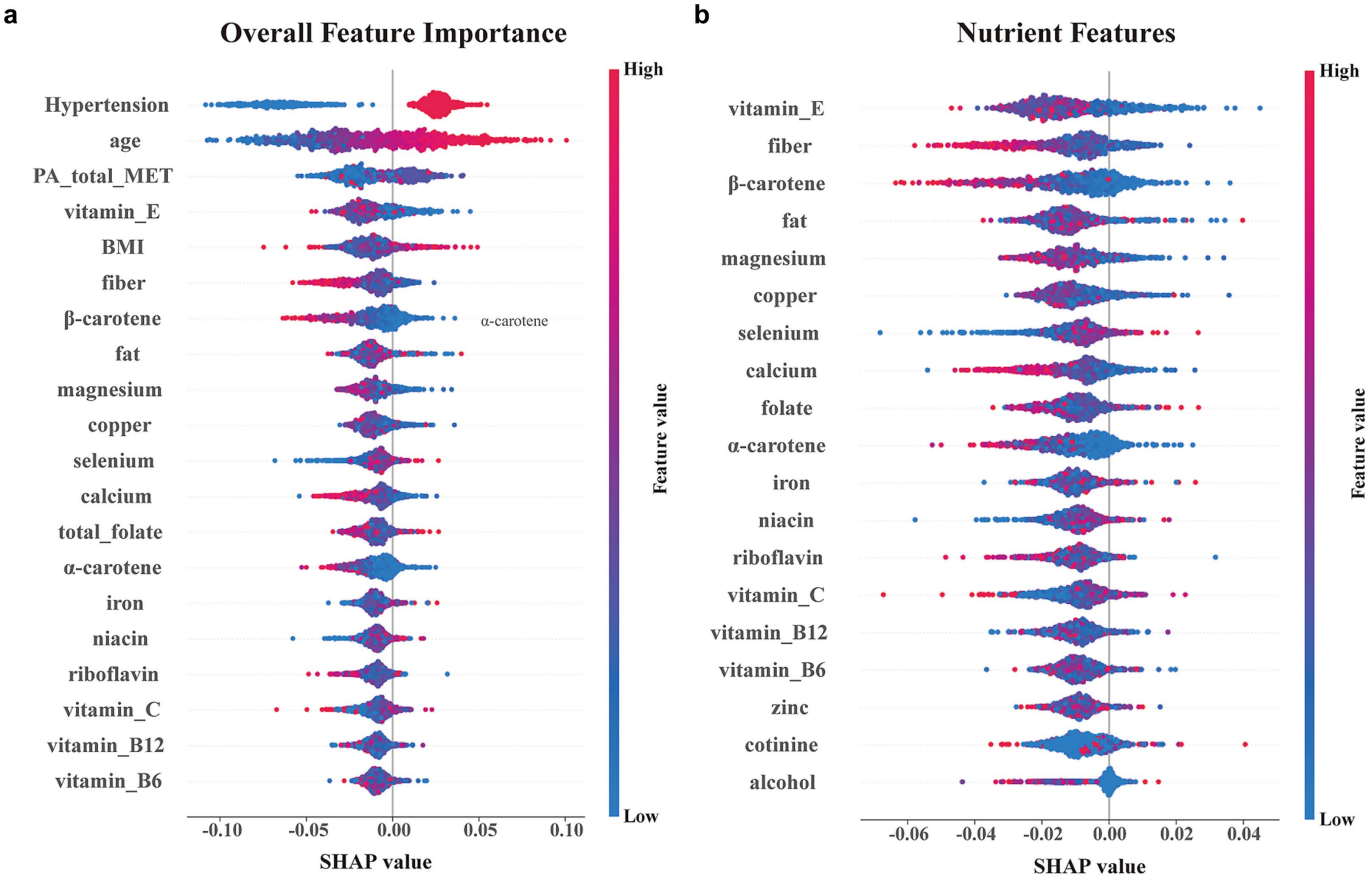
SHAP summary plots for overall and nutrient-specific feature importance. **(a)** Overall feature importance plot integrating demographic, lifestyle, and dietary variables. **(b)** Feature importance plot highlighting the contribution of oxidative balance-related dietary and exposure variables. Each point represents an individual sample, with color indicating feature value (red = high, blue = low), and the horizontal position reflecting the feature’s impact on the model output.

The SHAP analysis was performed to quantify the contribution of individual features to the random forest model (Figure 4). Among dietary factors, vitamin E, *β*-carotene, and magnesium ranked as the top contributors to model predictions. Higher values of vitamin E and β-carotene were predominantly associated with negative SHAP values, whereas magnesium showed a more variable contribution pattern.

**Figure 5:**
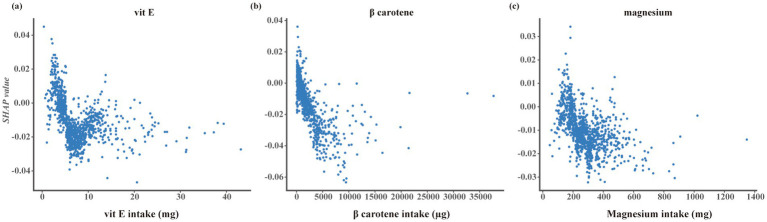
SHAP dependence plots for key nutrient features. SHAP dependence plots showing the relationships between nutrient intake levels and SHAP values for **(a)** vitamin E, **(b)** β-carotene, and **(c)** magnesium. The x-axis represents the intake level of each nutrient, and the y-axis represents the corresponding SHAP value for the prediction of UACR-defined renal impairment.

SHAP dependence plots further illustrated the relationships between nutrient intake levels and model output (Figure 5). Vitamin E and β-carotene demonstrated consistent negative SHAP values at higher intake levels, while magnesium exhibited a heterogeneous distribution across its intake range.

### 3.4 GO and KEGG analysis of nutrient-associated gene sets

**Figure 6:**
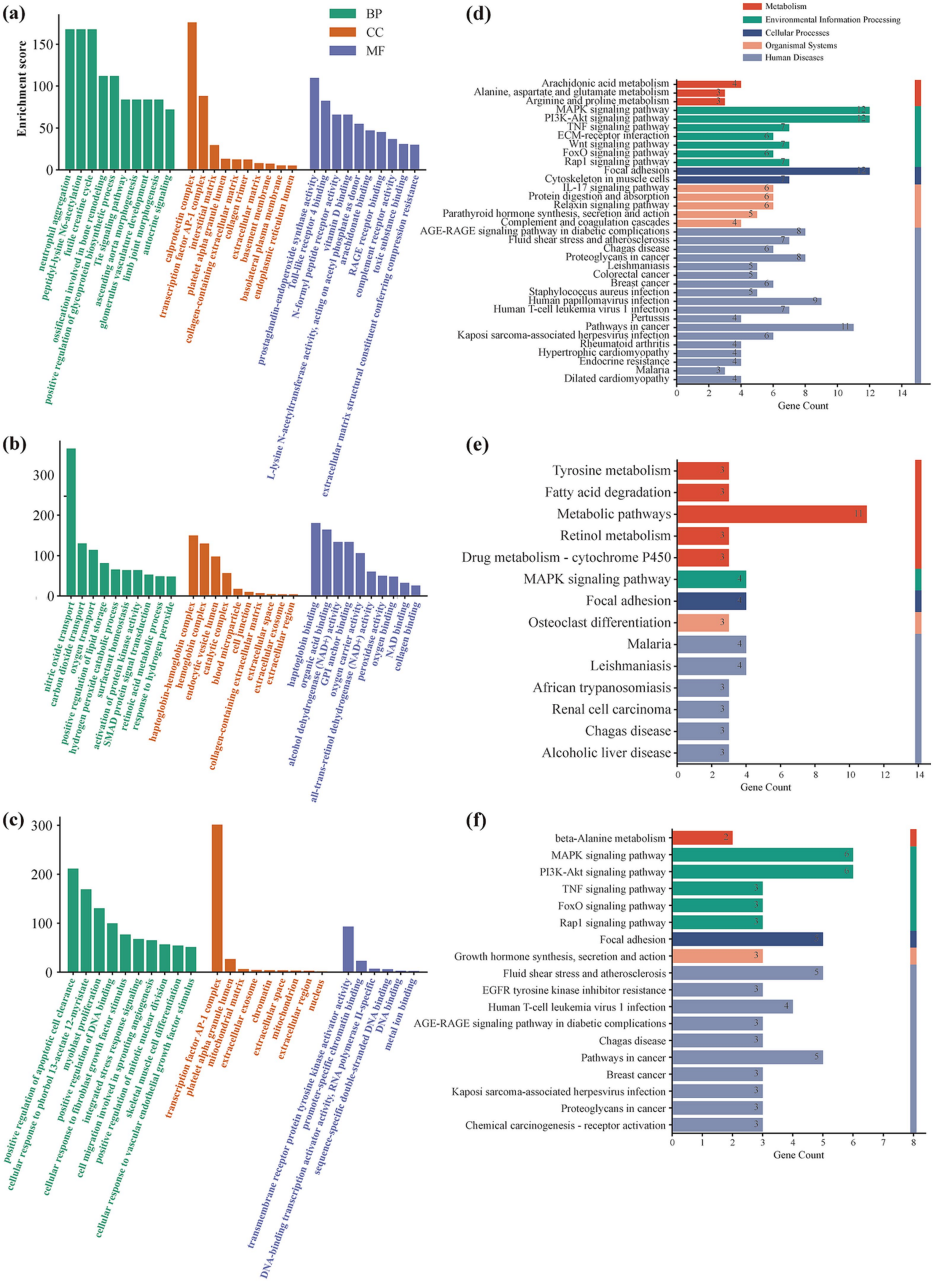
GO and KEGG enrichment analysis of nutrient-related gene sets. **(a–c)** GO enrichment results for genes associated with vitamin E **(a)**, β-carotene **(b)**, and magnesium **(c)**. The enriched terms are shown across three major categories: biological process (BP), cellular component (CC), and molecular function (MF). **(d–f)** KEGG pathway enrichment analysis corresponding to vitamin E **(d)**, β-carotene **(e)**, and magnesium **(f)**. The bar charts present significantly enriched pathways, including metabolism-related pathways, cellular processes, environmental information processing, and disease-associated pathways. Gene counts represent the number of genes involved in each pathway.

The GO and KEGG enrichment analyses were performed for gene sets associated with vitamin E, β-carotene, and magnesium to explore their functional characteristics related to renal impairment in diabetes (Figure 6).

For β-carotene–related genes, GO terms were primarily enriched in biological processes related to oxygen transport, lipid metabolism, and oxidative stress response, while KEGG analysis highlighted pathways including fatty acid degradation, retinol metabolism, cytochrome P450–related metabolism, MAPK signaling, and focal adhesion.

Magnesium-associated gene sets were enriched in GO terms related to apoptosis regulation, cell proliferation, DNA damage response, and mitochondrial function. KEGG pathway analysis identified enrichment in PI3K–Akt, MAPK, TNF, FoxO, and Rap1 signaling pathways, as well as focal adhesion and cytoskeletal regulation pathways.

Vitamin E–related targets showed enrichment in GO terms associated with metabolic processes, immune-related functions, and extracellular matrix organization. KEGG pathways included MAPK, PI3K–Akt, TNF, Wnt, FoxO, IL-17, Rap1 signaling, ECM–receptor interaction, and AGE–RAGE signaling.

### 3.5 Nutrient treatments modulate AKT phosphorylation in HK-2 cells

**Figure 7:**
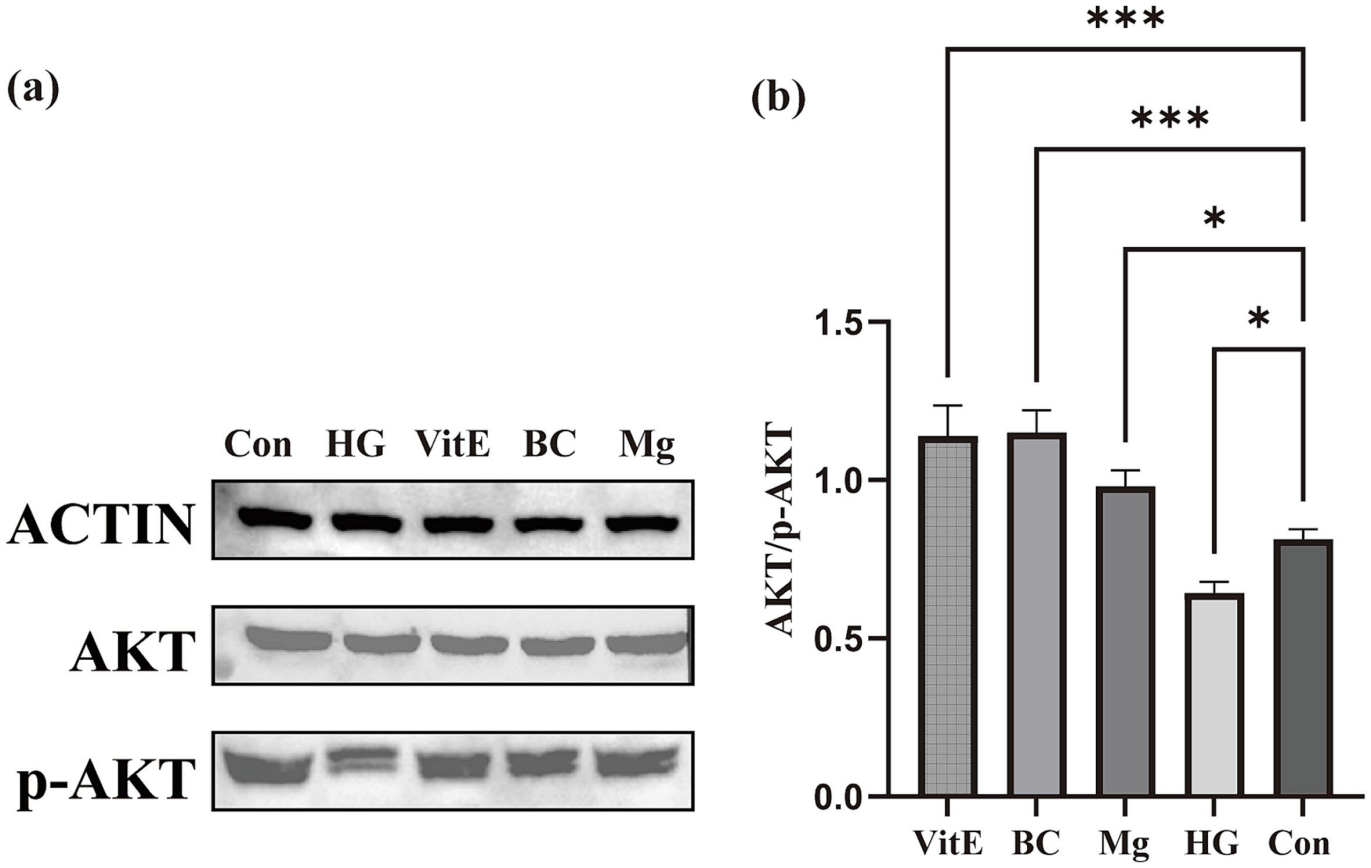
Effects of nutrient treatments on AKT phosphorylation in HK-2 cells. **(a)** Representative western blot images showing total AKT, phosphorylated AKT (p-AKT), and actin levels in HK-2 cells under different treatment conditions. Con, control; HG, high glucose; VitE, vitamin E; BC, β-carotene; Mg, magnesium treatment. **(b)** Quantification of AKT activation presented as the p-AKT/AKT ratio across treatment groups. High glucose significantly reduced AKT phosphorylation compared with the control, whereas vitamin E, β-carotene, and magnesium all partially restored AKT activation. Data are shown as mean ± SD from three independent experiments (*n* = 3). *p < 0.05, **p < 0.01, ***p < 0.001.

Given that the PI3K–Akt pathway was one of the significantly enriched pathways in the KEGG analysis, we focused the exploratory *in vitro* analysis on AKT phosphorylation as a downstream signaling readout relevant to high-glucose–associated cellular signaling changes in HK-2 cells. The effects of vitamin E, *β*-carotene, and magnesium on AKT phosphorylation were examined in HK-2 cells under high-glucose conditions (Figure 7). High glucose markedly reduced AKT phosphorylation compared with the control group. Treatment with vitamin E, β-carotene, or magnesium partially restored AKT phosphorylation, as reflected by increased p-AKT/AKT ratios relative to the high-glucose group. Among the three nutrients, vitamin E and magnesium showed a greater increase in AKT phosphorylation, while β-carotene exhibited a more moderate effect.

## 4 Discussion

This study systematically explored the complex relationship between oxidative balance-related dietary components and UACR-defined renal impairment in patients with diabetes using an interpretable machine learning approach. Compared with traditional linear regression or single-nutrient analyses (13–15), the random forest algorithm employed has advantages in handling high-dimensional and potentially nonlinear relationships. Furthermore, it may also better capture complex patterns among dietary factors and renal impairment that conventional statistical methods can overlook. Machine learning enables the simultaneous integration of 19 oxidative balance-related variables and 12 clinical covariates and provides a multidimensional analysis that may better reflect the complexity of nutritional and clinical influences in the human body. Notably, the SHAP (SHapley Additive exPlanations) interpretability analysis adopted in this study provides additional insights for nutritional epidemiology research and helps address the “black box” criticism often associated with machine learning models. This improves the interpretability of the findings while maintaining model performance. In addition, the SHAP-identified key nutrients informed our subsequent GO/KEGG enrichment analysis, and the significantly enriched PI3K-Akt pathway further guided the design of the exploratory *in vitro* experiments.

In the present study, the in vitro validation was designed to examine pathway-level signaling changes rather than to comprehensively quantify upstream oxidative stress markers. Although classical indicators such as ROS, SOD, and MDA are widely used to reflect oxidative status, the PI3K-Akt pathway was significantly enriched in our KEGG analysis and has also been implicated in kidney-related cellular stress pathways under diabetic conditions in previous studies. Therefore, AKT phosphorylation was assessed as a biologically relevant downstream readout to determine whether the identified antioxidant-related nutrients could modulate high-glucose–associated signaling changes in HK-2 cells. Nevertheless, the absence of direct oxidative stress measurements should be acknowledged, and future studies incorporating ROS, SOD, MDA, and related markers would help further strengthen the biological link to dietary oxidative balance.

Previous studies have suggested that magnesium, vitamin E, and β-carotene may be relevant to kidney-related phenotypes in diabetes. Magnesium, which is an essential mineral, plays critical roles in cellular signaling, energy metabolism, and antioxidant defense (16). Substantial evidence indicates that magnesium deficiency may contribute to chronic inflammation and oxidative stress (17), which is a pathological state closely linked to renal impairment and adverse renal phenotypes in diabetes. As early as the 1990s, studies revealed that low-magnesium diets were associated with nephrocalcinosis and renal dysfunction (18), while recent epidemiological data further confirmed that populations with higher magnesium intake exhibit significantly lower rates of chronic kidney disease (19). This association may be related to the multifaceted physiological functions of magnesium. Some of these functions are as follows: as a cofactor for numerous enzymes, it directly participates in insulin secretion and glucose metabolism regulation (20), and its deficiency has been identified as a risk factor for diabetes development (21). In the vascular system, magnesium may help alleviate diabetes-induced endothelial damage by modulating calcium channels, suppressing sympathetic activity, and lowering blood pressure (22). Notably, multiple observational studies have reported an association between low serum magnesium levels and adverse kidney-related measures, including faster decline in kidney function (23–25), particularly in patients with type 2 diabetes (24). These findings provide broader epidemiological context, although the present study focused specifically on cross-sectional UACR-defined renal impairment. Although the mechanisms linking hypomagnesemia to renal impairment remain incompletely understood, exacerbated oxidative stress, dysregulated inflammation, and accelerated vascular calcification have been proposed as possible contributors (26, 27). In this context, our SHAP analysis identified magnesium as an important contributor to the prediction model, supporting its possible relevance to UACR-defined renal impairment in diabetic populations (28). Consistent with these observations, our GO/KEGG enrichment analysis further indicated that magnesium-related targets were enriched in pathways involved in oxidative stress regulation and cellular survival signaling. However, establishing definitive causality requires rigorous intervention trials. Future mechanistic studies are needed to further clarify these pathways.

The potential role of vitamin E also warrants further discussion. The role of vitamin E, a classic lipid-soluble antioxidant, in the prevention of diabetes complications has been controversial (29, 30). However, our predictive model identified vitamin E as the top-weighted component among oxidative balance-related dietary factors. Prior clinical evidence, including a meta-analysis of randomized trials, has reported associations between vitamin E supplementation and changes in urinary albumin excretion in diabetic patients (SMD =0.33) (31). Mechanistically, chronic hyperglycaemia drives excessive reactive oxygen species (ROS) production and leads to podocyte injury and glomerular basement membrane thickening (32, 33). *In vitro* studies have shown that antioxidants, such as vitamin E, effectively neutralize these harmful free radicals and mitigate oxidative damage to renal tissue (34). Some clinical studies have also reported associations between vitamin E supplementation and albuminuria-related measures in diabetic patients with micro- or macroalbuminuria (35). Our GO/KEGG enrichment analysis further suggested that vitamin E–related targets were involved in pathways linked to oxidative stress regulation and metabolic homeostasis. However, significant heterogeneity exists across studies because of variations in dosage, formulation, treatment duration, and baseline patient characteristics (31), which underscores the need for further evaluation in different patient subgroups.

Among carotenoids, β-carotene has emerged as particularly influential. As one of the most abundant carotenoids in humans (36), β-carotene may help protect cells from oxidative damage via potent free radical scavenging (37). This antioxidant property may partly explain its inverse association with diabetic complications. Previous prospective studies have reported associations between higher circulating carotenoid levels and a lower risk of rapid kidney function decline (38). Although these studies addressed broader renal outcomes than the cross-sectional UACR-defined outcome used here, they provide contextual support for further investigation of β-carotene in diabetic populations. In addition, a 14-year follow-up study of 3,107 diabetic patients revealed that higher serum β-carotene levels significantly reduced cardiovascular mortality risk (39). These epidemiological observations intriguingly mirror our model’s results, where SHAP analysis revealed that β-carotene was the third most important predictor after vitamin E and magnesium. Consistent with these findings, our GO/KEGG enrichment analysis indicated that β-carotene–related targets were enriched in pathways associated with lipid metabolism and oxidative stress responses. Notably, increased dietary β-carotene intake is also associated with a reduced incidence of type 2 diabetes (40), which suggests that its potential beneficial associations may extend across different stages of the diabetes continuum. Given that carotenoid bioavailability depends on factors, such as dietary fat content and individual absorption differences, future studies should further examine the relationships of β-carotene status, intake patterns, and albuminuria-related phenotypes in diabetic populations.

From an observational perspective, our findings help highlight dietary factors associated with cross-sectional UACR-defined renal impairment in diabetes. The key nutrients identified—vitamin E, magnesium, and β-carotene—were among the dietary factors most strongly associated with UACR-defined renal impairment in our analytic sample. In exploratory analyses, GO/KEGG enrichment suggested that these nutrients were linked with pathways related to oxidative stress modulation and cellular survival. For instance, diabetic patients with lower serum magnesium (24, 41, 42) or carotenoid status (38) may represent subgroups of interest for future observational research. These findings may help guide future research on dietary patterns and nutrient-related phenotypes in diabetic populations with UACR-defined renal impairment. The identification of vitamin E, magnesium, and β-carotene as the most influential dietary factors may help prioritize these nutrients for further investigation in diabetic populations with UACR-defined renal impairment. In particular, foods rich in these nutrients—such as nuts, seeds, green leafy vegetables, and carrots—may warrant further investigation in future dietary studies, rather than being interpreted as evidence for risk reduction. Furthermore, future studies may examine whether these micronutrients help characterize nutritional profiles associated with UACR-defined renal impairment in diabetes.

Several limitations must be acknowledged. As this was an observational study based on NHANES data, the observed relationships should be interpreted as associations rather than causal effects, and residual confounding cannot be entirely ruled out. Importantly, the outcome in this study was cross-sectional UACR-defined renal impairment rather than longitudinal kidney function decline or diabetic nephropathy progression. Although machine learning models excel at capturing complex nonlinear relationships, dietary data collected via 24-h recall methods are susceptible to measurement errors. Additionally, the absence of direct oxidative stress biomarkers (e.g., ROS levels or antioxidant enzyme activity) (33) limits mechanistic and causal inferences. Nevertheless, our experimental validation in HK-2 cells, which demonstrated nutrient-associated restoration of AKT phosphorylation under high-glucose conditions, provides limited pathway-level support for the biological plausibility of the observed associations. Future studies should integrate multiomics data (e.g., genomics and metabolomics) to elucidate the intricate links between oxidative balance-related variables and renal impairment in diabetes.

Despite the moderate predictive performance of our model (AUC ≈ 0.73), it is important to emphasize that such a level of discrimination is not uncommon in nutritional epidemiology studies involving multifactorial outcomes such as renal impairment in diabetes. The complex interplay between dietary components, lifestyle factors, and clinical covariates inherently limits the achievable AUC in risk prediction models based solely on questionnaire-derived and demographic data. Rather than focusing solely on classification accuracy, the primary strength of this study lies in its interpretable machine learning framework, which leverages SHAP values to identify and rank key dietary factors—most notably vitamin E, magnesium, and β-carotene—that were associated with renal impairment in diabetic patients. These SHAP-derived nutrient signatures were further explored through our subsequent GO/KEGG pathway enrichment analysis and functional validation experiments, reinforcing the biological plausibility of the model’s predictions.

Furthermore, our use of a large sample from NHANES, rigorous preprocessing, and a transparent analytical workflow enhance the reliability of our findings. Nevertheless, we acknowledge that the inclusion of additional biomarkers—such as genetic variants, metabolomic profiles, or direct measures of oxidative stress (e.g., ROS levels, antioxidant enzyme activities)—could improve model performance in future work. Integrating multi-omics data may not only enhance predictive accuracy but also provide additional biological context for the associations between oxidative balance-related variables and renal impairment in diabetes. In this context, our exploratory experiments demonstrating nutrient-associated restoration of AKT phosphorylation offer exploratory pathway-level support for the biological plausibility of the observed associations.

Future research should include prospective studies, mechanistic investigations, and external validation analyses to clarify the roles of magnesium, vitamin E, and β-carotene in diabetic renal phenotypes (28, 31).

## 5 Conclusion

This study employs advanced machine learning to identify associations between oxidative balance-related dietary factors and renal impairment in diabetes. The identification of vitamin E, magnesium, and β-carotene as key predictors highlights dietary factors associated with UACR-defined renal impairment in diabetes and may inform future observational and mechanistic research. Moreover, the consistency between model-derived nutrient signatures, exploratory enrichment results, and AKT-related experimental findings offers exploratory pathway-level support for the biological plausibility of these associations. Future studies may further examine heterogeneity in nutrient-related profiles among diabetic patients with UACR-defined renal impairment.

## Data Availability

Publicly available datasets were analyzed in this study. The datasets analyzed are publicly available from the NHANES database. The analysis code is publicly available at: https://github.com/noctunroscara-ui/NHANES-OBS-ML-Renal-Impairment.
