# Supplementary material for: Dietary oxidative balance and renal impairment in diabetes identified by machine learning and functional analysis
Source: Front Nutr. 2026 May 4;13:1792300. doi: 10.3389/fnut.2026.1792300 (PMC13180596; doi:10.3389/fnut.2026.1792300)
Supplement: Supplementary file 1 [file Supplementary_file_1.docx]

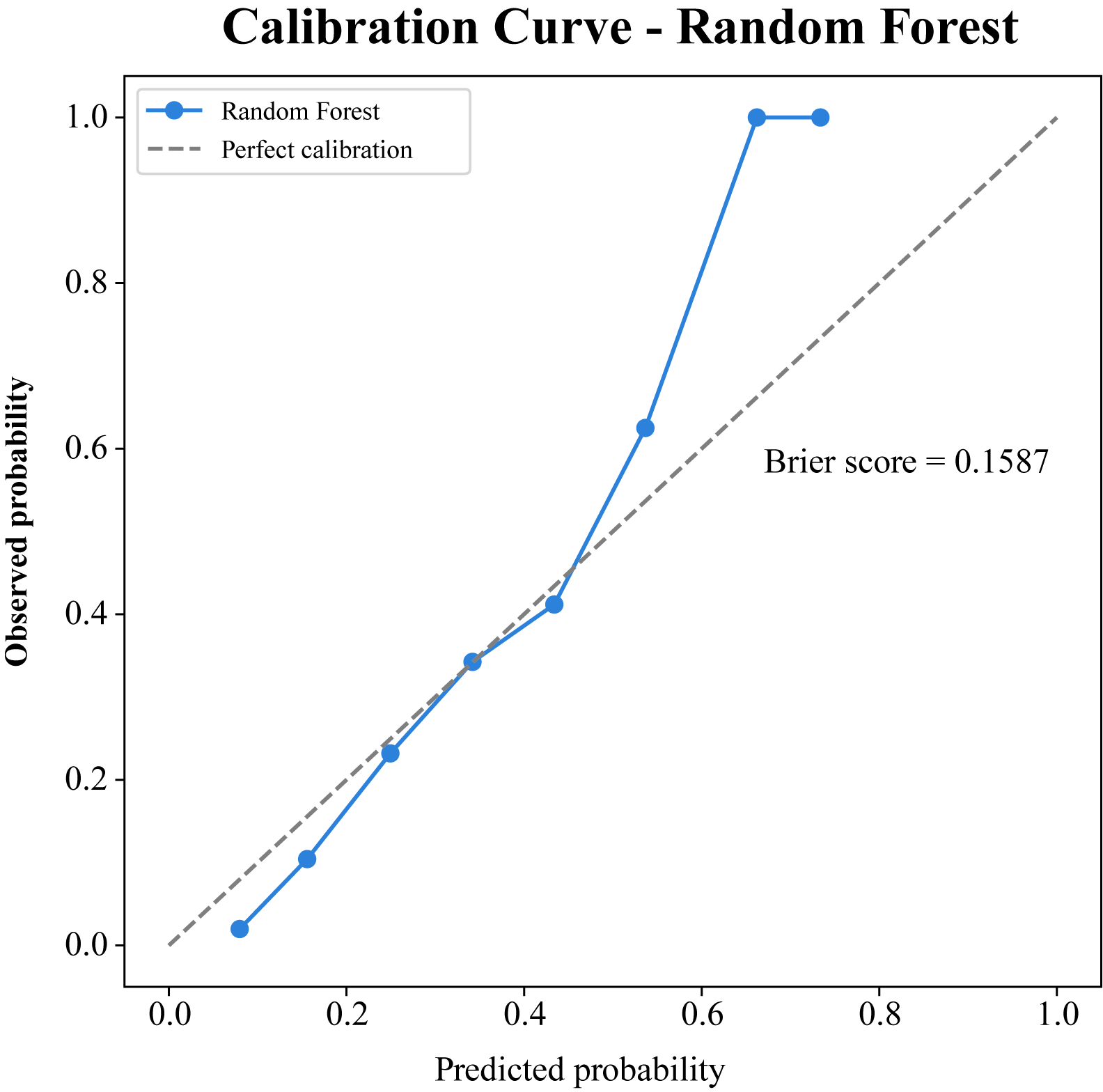


**Supplementary Figure 1. Calibration curve of the random forest model.** Calibration plot of the random forest model showing the relationship between predicted and observed probabilities. The dashed diagonal line indicates perfect calibration. Some deviation from the ideal line was observed at higher predicted probabilities. The Brier score was 0.1587.
