# Supplementary material for: Dietary oxidative balance and renal impairment in diabetes identified by machine learning and functional analysis
Source: Front Nutr. 2026 May 4;13:1792300. doi: 10.3389/fnut.2026.1792300 (PMC13180596; doi:10.3389/fnut.2026.1792300)
Supplement: Supplementary file 2 [file Table_1.docx]

| oxidative balance-related variables | | | | |
| --- | --- | --- | --- | --- |
| Dietary fiber | Total fat | α-carotene | β-carotene | Riboflavin |
| Niacin | Vitamin B6 | Total folate | Vitamin B12 | Vitamin C |
| Vitamin E | Calcium | Magnesium | Iron | Zinc |
| Selenium | Alcohol | Cotinine |  |  |

**Supplementary Table 1. Oxidative balance-related variables included in the analysis.**

| **Variable** | **Overall  N = 9,764^1^** | **without kidney impairment, N = 7,420^1^** | **with kidney impairment, N = 2,344^1^** | **p-value^2^** |  |
| --- | --- | --- | --- | --- | --- |
| **fiber** | 14.70 (10.30, 20.60) | 15.10 (10.45, 21.10) | 13.85 (9.88, 19.25) | <0.001 |  |
| **fat** | 69.01 (48.61, 94.96) | 69.93 (49.17, 95.76) | 66.36 (46.40, 91.83) | <0.001 |  |
| **α-carotene** | 91.00 (26.50, 431.75) | 95.50 (27.00, 444.00) | 79.50 (24.75, 375.75) | 0.003 |  |
| **β-carotene** | 1,106.25 (434.50, 2,710.50) | 1,162.75 (451.50, 2,793.50) | 970.50 (379.25, 2,445.25) | <0.001 |  |
| **riboflavin** | 1.78 (1.29, 2.37) | 1.79 (1.30, 2.40) | 1.74 (1.26, 2.30) | <0.001 |  |
| **niacin** | 21.27 (15.40, 28.30) | 21.49 (15.58, 28.62) | 20.27 (14.77, 27.49) | <0.001 |  |
| **vitamin_B6** | 1.69 (1.21, 2.31) | 1.71 (1.23, 2.34) | 1.60 (1.15, 2.23) | <0.001 |  |
| **total_folate** | 334.50 (240.00, 457.00) | 338.00 (244.50, 463.00) | 320.25 (230.00, 435.00) | <0.001 |  |
| **vitamin_B12** | 3.80 (2.40, 5.89) | 3.84 (2.43, 5.90) | 3.66 (2.31, 5.81) | 0.034 |  |
| **vitamin_C** | 61.35 (30.18, 109.30) | 62.40 (30.40, 110.23) | 58.85 (29.70, 106.65) | 0.020 |  |
| **vitamin_E** | 6.45 (4.38, 9.22) | 6.59 (4.52, 9.32) | 5.91 (4.08, 8.92) | <0.001 |  |
| **calcium** | 768.00 (535.00, 1,061.75) | 781.50 (545.00, 1,077.25) | 725.00 (505.25, 1,001.50) | <0.001 |  |
| **magnesium** | 258.00 (194.00, 337.25) | 261.50 (197.00, 342.25) | 242.50 (184.00, 322.00) | <0.001 |  |
| **iron** | 12.79 (9.36, 17.38) | 12.98 (9.49, 17.51) | 12.29 (9.09, 16.93) | <0.001 |  |
| **zinc** | 9.52 (6.84, 13.17) | 9.65 (6.93, 13.25) | 9.09 (6.60, 12.89) | <0.001 |  |
| **copper** | 1.07 (0.81, 1.42) | 1.08 (0.82, 1.44) | 1.00 (0.77, 1.34) | <0.001 |  |
| **selenium** | 98.68 (71.80, 130.78) | 99.78 (72.45, 131.55) | 96.05 (69.90, 128.63) | 0.003 |  |
| **alcohol** | 0.00 (0.00, 0.00) | 0.00 (0.00, 0.00) | 0.00 (0.00, 0.00) | <0.001 |  |
| **cotinine** | 0.04 (0.01, 0.75) | 0.04 (0.01, 0.60) | 0.04 (0.01, 1.52) | 0.057 |  |
| ^1^Median (Q1, Q3) | | | | |  |
| ^2^Wilcoxon rank sum test | | | | |  |

**Supplementary Table 2. Baseline characteristics of participants according to renal impairment status defined by UACR.** Continuous variables are presented as median (interquartile range, IQR), and categorical variables are presented as number (percentage). Differences between groups were assessed using the Wilcoxon rank-sum test for continuous variables and the chi-square test for categorical variables.

| **Model** | **AUC (95% CI)** | **PR AUC** | **Sensitivity** | **Specificity** | **F1 Score** | **Balanced Accuracy** |
| --- | --- | --- | --- | --- | --- | --- |
| **Random Forest** | 0.7205 (0.6934–0.7461) | 0.4970 | 0.7164 | 0.5856 | 0.4732 | 0.6510 |
| **XGBoost** | 0.7009 (0.6739–0.7257) | 0.4108 | 0.6951 | 0.5923 | 0.4657 | 0.6437 |
| **Gradient Boosting** | 0.6978 (0.6712–0.7240) | 0.4063 | 0.7100 | 0.5903 | 0.4723 | 0.6502 |
| **SVM** | 0.6938 (0.6655–0.7194) | 0.3991 | 0.5778 | 0.7136 | 0.4652 | 0.6457 |
| **Logistic Regression** | 0.6738 (0.6466–0.7008) | 0.3937 | 0.7335 | 0.5142 | 0.4485 | 0.6238 |

**Supplementary Table 3. Comparison of predictive performance among five machine learning models for renal impairment in diabetes.** The predictive performance of random forest, XGBoost, gradient boosting, support vector machine, and logistic regression was evaluated on the independent test set. Reported metrics include AUC with 95% confidence intervals, PR AUC, sensitivity, specificity, F1 score, and balanced accuracy.
